# Multi-center implementation of rapid whole genome sequencing provides additional evidence of its utility in the pediatric inpatient setting

**DOI:** 10.3389/fped.2024.1349519

**Published:** 2024-02-19

**Authors:** Lauren Thompson, Austin Larson, Lisa Salz, Regan Veith, John-Paul Tsai, Anuj Jayakar, Rachel Chapman, Apeksha Gupta, Stephen F. Kingsmore, David Dimmock, Alan Bedrick, Maureen Kelly Galindo, Kari Casas, Mohamed Mohamed, Lisa Straight, M. Akram Khan, Daria Salyakina

**Affiliations:** ^1^ Division of Genetics and Metabolism, Nicklaus Children’s Hospital, Miami, FL, United States; ^2^ Department of Pediatrics, Children’s Hospital Colorado, Aurora, CO, United States; ^3^ Department of Pediatrics, University of Colorado School of Medicine, Aurora, CO, United States; ^4^ RCIGM, Rady Children’s Hospital San Diego, San Diego, CA, United States; ^5^ Genomic Medicine, Children’s Minnesota, Minneapolis, MN, United States; ^6^ Fetal & Neonatal Institute, Children’s Hospital Los Angeles, Los Angeles, CA, United States; ^7^ Department of Pediatrics, USC Keck School of Medicine, Los Angeles, CA, United States; ^8^ Department of Pediatrics, Banner Diamond Children’s Medical Center, Tucson, AZ, United States; ^9^ Department of Pediatrics, University of Arizona College of Medicine, Tucson, AZ, United States; ^10^ Department of Pediatrics, Sanford Children’s Fargo, Fargo, SD, United States; ^11^ Department of Pediatrics, Sanford Children’s Sioux Falls, Sioux Falls, SD, United States

**Keywords:** genomic testing, diagnostic testing, rare disease, neonatal, pediatric

## Abstract

**Objective:**

Multi-center implementation of rapid whole genome sequencing with assessment of the clinical utility of rapid whole genome sequencing (rWGS), including positive, negative and uncertain results, in admitted infants with a suspected genetic disease.

**Study design:**

rWGS tests were ordered at eight hospitals between November 2017 and April 2020. Investigators completed a survey of demographic data, Human Phenotype Ontology (HPO) terms, test results and impacts of results on clinical care.

**Results:**

A total of 188 patients, on general hospital floors and intensive care unit (ICU) settings, underwent rWGS testing. Racial and ethnic characteristics of the tested infants were broadly representative of births in the country at large. 35% of infants received a diagnostic result in a median of 6 days. The most common HPO terms for tested infants indicated an abnormality of the nervous system, followed by the cardiovascular system, the digestive system, the respiratory system and the head and neck. Providers indicated a major change in clinical management because of rWGS for 32% of infants tested overall and 70% of those with a diagnostic result. Also, 7% of infants with a negative rWGS result and 23% with a variant of unknown significance (VUS) had a major change in management due to testing.

**Conclusions:**

Our study demonstrates that the implementation of rWGS is feasible across diverse institutions, and provides additional evidence to support the clinical utility of rWGS in a demographically representative sample of admitted infants and includes assessment of the clinical impact of uncertain rWGS results in addition to both positive and negative results.

## Introduction

Genetic disorders are a leading cause of admissions to the neonatal intensive care unit (NICU) in the United States (1–3). With improving technology and access to genetic testing, rapid whole genome sequencing (rWGS) has become an important diagnostic tool for critically ill infants with potential undiagnosed genetic disorders.

Whole genome sequencing (WGS) is a comprehensive genetic diagnostic test with broad sensitivity for most known single locus (Mendelian) disorders (4–6). WGS has a greater diagnostic yield compared to targeted gene panels and it can detect diagnostic results beyond the scope of whole exome sequencing, including intronic and extra-genic variants, and exonic single nucleotide variants (SNVs) missed due to poor sequencing read depth with exome capture (7, 8). In addition, WGS can identify large and small copy number and structural variants with higher sensitivity than a microarray and can detect mitochondrial DNA mutations (6, 9, 10). Rapid whole genome sequencing (rWGS) typically refers to reporting of results within five to seven days of sample collection, a timeframe that is relevant for impacting medical decision making within an inpatient hospitalization (7, 11).

There have been multiple single- and multi-center studies focusing on understanding the diagnostic yield, clinical utility, and financial impact of WGS in a variety of clinical contexts including the intensive care unit and neonatal populations. Notable studies on these topics include BabySeq, Baby Bear, Baby Manatee, GEMINI, Baby Deer, NICUSeq, NSIGHT, NSIGHT2 and SouthSeq (2, 7, 12–18). Results of those studies have generally demonstrated relatively high diagnostic yield, clinical utility and cost effectiveness for WGS and rWGS.

In this paper, we further characterize the impact of rWGS, reporting on the clinical impact and the specific genetic diagnoses found for infants admitted at eight different participating sites with diverse patient populations and varying composition of clinical teams employing rWGS.

## Methods

Singleton (proband), parent-child duo, or parent-child trio rWGS tests (Supplementary Table S1) were ordered through Rady Children's Institute for Genomic Medicine between November 2017 and April of 2020. Inclusion criteria were infants admitted to the hospital (either intensive care unit or general floor) who were less than 1 year of age, had a suspected genetic condition, and received rWGS. There were no specified exclusion criteria and individuals with or without prior genetic testing were included with the exception of those with a clear diagnostic result from prior testing. Appropriate consent was obtained by each site. Sequencing was done per a previously published rWGS protocol (7, 13, 14, 17). Briefly, DNA was extracted from whole blood, sonicated and sequencing libraries were performed without PCR. Sequencing was performed to at least 40× read depth using Illumina sequencers. Sequence and structural variants were called using separate bioinformatic pipelines and then variants were filtered and analyzed by molecular geneticists. Secondary findings were not systematically sought or reported, but medically actionable incidental findings were reported if families consented to receiving this information. Verbal preliminary results were provided to the ordering physician by a lab director when considered to have immediate implications for management, followed by written preliminary reports and ultimately final confirmed results. The final report was considered a clinical test result and was included in the electronic health record for the patient. Once completed, one clinician from each site reviewed medical records for rWGS cases and completed a survey documenting the characteristics of tested patients, genetic results and their assessment of the impact of rWGS testing on the care of the infant. Such clinicians varied by site and included geneticists, genetic counselors, neonatologists, a cardiologist, a neurologist and a pediatric intensivist.

### Study sites

Eight hospitals participated in the study including Children's Minnesota (Minneapolis, MN), Nicklaus Children's (Miami, FL), Rady Children's (San Diego, CA), Children's Colorado (Aurora, CO), Banner Diamond Children's Medical Center, Tucson, AZ (Tucson, AZ), Sanford Children's Fargo (Fargo, ND), Sanford Children's Sioux Falls (Sioux Falls, SD) and Children's Hospital Los Angeles (Los Angeles, CA). Each hospital had their own institutional research protocol for prospective enrollment or retrospective analysis of clinical data. Each site was provided with ten rWGS tests to be used according to local discretion. However, some sites did not use all of their testing while others had additional funding available. Therefore, the number of tests used varied between 3 and 58 based on the site itself (Supplementary Table S2).

### Patient data collection

A clinician at each site completed a survey about each included subject. Demographic data included age at testing, gestational age at birth, sex, race, and ethnicity. Gestational age was categorized as extreme preterm (<28 weeks), very preterm (28–31 weeks), moderate-late preterm (32–36 weeks), and full term (≥37 weeks) according to the commonly used definitions of these categories (19). Age at testing was categorized as ≤28 days and >28 days after birth, corresponding to the commonly used definition of the neonatal period.

Clinical genetic results were categorized as primary diagnosis (identification of the genetic etiology of the phenotype that was the indication for testing), an incidental diagnosis (a reportable genetic diagnosis not related to the indication for testing), negative results, and variants of unknown significance (VUS) on the basis of the assertion of result type from the reference laboratory. Cases with multiple result types on rWGS were hierarchically categorized as (1) diagnostic if at least one primary diagnostic result was reported regardless of other result types present; (2) VUS if both VUS and incidental results were present; (3) incidental result if neither diagnostic nor VUS results were present in addition to the incidental results; (4) negative if none of the three result types were reported.

The following impacts were documented based on the assessment of the medical records by site investigators: changes in medical management, clinical assessment of global impact of rWGS on health outcomes (better, worse, no change), likely change in length of stay (shorter, no change, longer, patient discharged prior to test results, patient died prior to test results). Specific clinical management impacts of rWGS were documented including changes in use of medications, surgeries, diet, organ transplant, other diagnostic evaluations, specialty consultations, screening regimen, referral for palliative care, referral for additional genetic counseling, and genetic testing for other family members (Supplementary Table S3). These different management changes were grouped into major, minor, or no change. Major changes in management were defined as changes in the use of medication, diet, surgery, or palliative care based on the rWGS results as these were determined by the research team to be potentially disease modifying. Minor management changes were defined as implementation of additional genetic counseling to discuss recurrence risk and family planning options, additional workup or consultation for the patient or genetic testing for additional family members. Cases with multiple types of management changes were categorized as major if both major and minor changes were identified.

Patient clinical presentation was translated into Human Phenotype Ontology (HPO) terms. HPO terms were assigned by the testing laboratory for genomic analysis based on medical records provided at the time of testing. Specific HPO terms were mapped back to organ system level HPO terms for all cases. Two HPO terms of organ systems were reclassified for this analysis, based on the authors’ consensus of the most accurate representation of the usage of these terms in the context of admitted neonates and infants. Specifically, elevated conjugated bilirubin was changed from metabolic to digestive organ system (reflecting the typical indication for testing being concern for hepatopathy) and hypotonia was changed from muscle organ system to neurological (reflecting the etiology of hypotonia in infancy).

### Statistical analysis

Descriptive analysis was used for the variables in the dataset. Means with standard deviations were reported for continuous data and counts with proportions were reported for categorical variables. *P*-values for categorical variables were reported using Fisher's exact test. *P*-values of <0.05 were used as the threshold for statistical significance. All the analyses were completed in SAS 9.4 (SAS Institute Inc., Cary, NC, USA) statistical software.

## Results

### Patients enrolled

A total of 188 admitted infants received rWGS testing across the eight sites between July 2017 and August 2020. 60% of infants tested were male and 40% were female. Racial and ethnic characteristics of the tested infants were broadly representative of births in the United States (Table 1), with a slight overrepresentation of Hispanic ethnicity (26% nationwide in a recent birth cohort, 31% in this study, *p*-value = 0.12) and a slight underrepresentation of Black race (16% nationwide in a recent birth cohort, 9% in this study, *p*-value = 0.008) (20). The median age at testing was 25 days old. Gestational age category at birth was full term for 73% of those tested, late preterm for 17%, very preterm for 6% and extreme preterm for 4%.

**Table 1:** Patients’ demographics.

Gender	*N* (%)
Male	112 (59.6)
Female	76 (40.4)
Race	
White	125 (66.5)
Other race	20 (10.6)
Black African American	17 (9.0)
Mixed race	10 (5.3)
Asian	9 (4.8)
Native Hawaiian or Other Pacific Islander	5 (2.7)
American Indian or Alaska Native	2 (1.1)
Ethnicity	
Non-Hispanic	127 (67.6)
Hispanic	59 (31.4)
No information	2 (1.1)
Gestational age	
Full term^a^	138 (73.4)
Late preterm	31 (16.5)
Very preterm	11 (5.9)
Extreme preterm	8 (4.3)

^a^ Full term is defined by >37 weeks gestation.

Phenotype-driven diagnostic interpretation of rWGS data was performed based on a single HPO term for 31 infants (16%) and on multiple HPO terms for 157 infants (84%). The rate of diagnostic results for infants with a single HPO term was similar to the overall rate of diagnosis (9/31 or 29%). For 61% of infants, the phenotype included an abnormality of the nervous system, 44% had an abnormality of the cardiovascular system, 38% had an abnormality of the digestive system, 30% had an abnormality of the respiratory system, 27% had an abnormality of the head and neck.

### Rate of diagnosis

Overall, 35% of infants had a diagnostic result, 49% had a negative result, 12% had a VUS and 4% had an incidental finding (Table 2). For very premature and extreme premature infants (*n* = 19), 16% had a diagnostic result as compared to 37% for those born at 32 weeks gestation and later (*n* = 169) (*p* = 0.06). For infants tested in the neonatal period (*n* = 107), 34% had a diagnostic result as compared to 37% for those infants tested after neonatal period (*n* = 81) (*p* = 0.67).

**Table 2:** Clinical changes in patients receiving rWGS.

Characteristics	Overall (%)	Diagnostic category			
		Negative	Diagnostic result	VUS	Secondary diagnosis
	*N* (%)	*N* (%)	*N* (%)	*N* (%)	*N* (%)
Total	188 (100.0)	93 (49.0)	66 (35.0)	22 (12.0)	7 (4.0)
Gender					
Male	112 (59.6)	46 (24.5)	45 (23.9)	17 (9.0)	4 (2.1)
Female	76 (40.4)	47 (25.0)	21 (11.2)	5 (2.7)	3 (1.6)
Global change					
No change	103 (54.8)	68 (36.2)	17 (9.0)	12 (6.4)	6 (3.2)
Better	80 (42.6)	24 (12.8)	47 (25.0)	8 (4.3)	1 (0.5)
Worse	5 (2.7)	1 (0.5)	2 (1.1)	2 (1.1)	0 (0.0)
Stay length change					
No change	133 (70.7)	83 (44.1)	27 (14.4)	16 (8.5)	7 (3.7)
Shorter	37 (19.7)	3 (1.6)	30 (16.0)	4 (2.1)	0 (0.0)
Patient discharged prior to test results	9 (4.8)	3 (1.6)	4 (2.1)	2 (1.1)	0 (0.0)
Longer	7 (3.7)	3 (1.6)	4 (2.1)	0 (0.0)	0 (0.0)
Patient died prior to test results	2 (1.1)	1 (0.5)	1 (0.5)	0 (0.0)	0 (0.0)
Gestation age (weeks)^a^					
Full term	138 (73.4)	65 (34.6)	52 (27.7)	17 (9.0)	4 (2.1)
Preterm	50 (26.6)	28 (14.9)	14 (7.4)	5 (2.7)	3 (1.6)
Management change^b^					
Minor	76 (40.4)	47 (25.0)	18 (9.6)	10 (5.3)	1 (0.5)
Major	61 (32.4)	6 (3.2)	46 (24.5)	5 (2.7)	4 (2.1)
No change	51 (27.1)	40 (21.3)	2 (1.1)	7 (3.7)	2 (1.1)
Testing age (days)					
≤28 days	107 (56.9)	53 (28.2)	36 (19.1)	14 (7.4)	4 (2.1)
>28 days	81 (43.1)	40 (21.3)	30 (16.0)	8 (4.3)	3 (1.6)

rWGS, rapid whole genome squencing; VUS, variant of unknown significance.

^a^ Gestation age (weeks) was defined as <36 preterm, ≥37 full term.

^b^ Managment changes was defined hierarchically if any patient had any major change along with minor or other change then they were categorized as having major change, followed by only minor, and no change. Please refer to Appendix 1 to see which categories were categorized as major, minor, and no change.

There was a statistically significant difference in the diagnostic category of results between male and female infants (24% vs. 11%, *p* = 0.03). There were eight X chromosome diagnoses made in males and one in a female infant (not including incidental findings). Males comprised 60% of the tested infants and accounted for 68% of the diagnostic results. Details of all diagnostic results are in Supplementary Table S3. Our study did not find any statistically significant difference in rate of diagnosis or management change based on the phenotype reported as the indication for rWGS (Supplementary Tables S4, S5).

Diagnostic rWGS results were reported by the laboratory to the ordering provider in a median of 6 days after sample accessioning. Negative rWGS results were reported in a median of 7 days. VUS results were returned after a median of 8 days.

### Changes in management

In the opinion of the primary investigator at each site, 32% of all infants tested had a major change in medical management because of rWGS, 40% had a minor change in management and 27% had no change in management (Table 3). Among the patients that were either very premature or extremely premature (*n* = 19), 26% had a major change in management (Table 3). Among infants that had rWGS at or before 28 days of life (*n* = 107), 28% had a major change in management as compared to 38% among those that were tested after 28 days of age (*n* = 81, *p* = 0.14) (Supplementary Table S6). The fraction of infants with changes in management due to rWGS did not significantly differ based on gestational age or phenotypic category (Table 3; Supplementary Tables S5, S6).

**Table 3:** Sequencing results and management change stratified by gestational age.

		Gestational age^a^			
	All (*N* = 188)	<28 weeks (*N* = 11)	28–31 weeks (*N* = 8)	32–36 weeks (*N* = 31)	≥37 weeks (*N* = 138)
	*N* (%)	*N* (%)	*N* (%)	*N* (%)	*N* (%)
rWGS result category^b^					
Diagnostic	66 (35.1)	2 (18.2)	1 (12.5)	11 (35.5)	52 (37.7)
VUS	22 (11.7)	1 (9.1)	0 (0.0)	4 (12.9)	17 (12.3)
Secondary	7 (3.7)	2 (18.2)	0 (0.0)	1 (3.2)	4 (2.9)
Negative	93 (49.5)	6 (54.5)	7 (87.5)	15 (48.4)	65 (47.1)
Management change^c^					
Major	61 (32.4)	4 (36.4)	1 (12.5)	8 (25.8)	48 (34.8)
Minor	76 (40.4)	3 (27.3)	2 (25.0)	13 (41.9)	58 (42.0)
No change	51 (27.1)	4 (36.4)	5 (62.5)	10 (32.3)	32 (23.2)

rWGS, rapid whole genome squencing; VUS, variant of unknown significance.

^a^ Gestation age (weeks) was defined as <36 preterm, ≥37 full term.

^b^ Category was defined hierarchically if any patient had diagnosis along with VUS, secondary diagnosis, or other change then they were categorized as having diagnositc, followed by only VUS, secondary, and negative.

^c^ Managment changes was defined hierarchically if any patient had any major change along with minor or other change then they were categorized as having major change, followed by only minor, and no change. Please refer to Appendix 1 to see which categories were categorized as major, minor, and no change.

Of the infants that received a diagnostic result, 97% had a resultant change in management, of which 70% were major changes in management and 27% were minor (Table 2). Of the infants who received a VUS result, 68% had a resultant change in management, of which 23% had a major change in management and 45% were minor (Table 2). Of the infants that received a negative rWGS result, 58% had a consequent change in management, of which 7% had a major change in management and 51% were minor (Table 2). Of seven infants that received an incidental finding, five (71%) had a consequent change in management, of which four were major (Table 2).

### Global impression

Investigators judged that 43% of patients had overall improved clinical care based on having received rWGS, 55% had no change and 3% of infants had worse clinical care due to rWGS (Table 2). Site investigators assessed that 20% of patients were likely to have had a shorter hospital stay because of rWGS, 71% were likely to have had no change in length of stay and 4% were likely to have had longer length of admission; 5% of patients were discharged prior to test results and 2% died prior to test results (Table 2).

An investigator assessment of likely shorter stay based on having had rWGS was significantly correlated with diagnostic vs. nondiagnostic results (Table 2). There was also a statistically significant correlation between investigator assessment of global improvement due to rWGS and diagnostic result as opposed to negative result (*P*-value is <0.0001).

### Genetic diagnoses identified

See Supplementary Table S3 for all the diagnostic rWGS results.

## Discussion

Our study provides documentation of successful multi-center implementation of rWGS and supportive evidence for the utility of rWGS in hospitalized infants with suspected genetic conditions alongside many other notable studies including BabySeq, Project Baby Bear, Baby Manatee, GEMINI, NICUSeq, Baby Deer, SouthSeq, NSIGHT1 and NSIGHT2. A distinctive feature of our study is its racial, ethnic, and geographical diversity, broadly representative of the United States population of infants. An additional distinctive feature of this study was the assessment of the clinical impact of VUSs.

188 patients, with a slight male predominance, were included in our study. This is a comparable number of participants, with similar gender differences, to other studies (7, 14).The diagnostic yield was higher in males than females, but the difference between sexes is not significant after removing X-linked recessive disorders. Regarding racial and ethnic distribution, prior studies such as NICUSeq and GEMINI, had an overrepresentation of white race, with 70% and 58% respectively (2, 7). In contrast, SouthSeq focused on racial/ethnic minorities and rural, medically underserved, areas with a higher African American population at 34% (18). Our study was broadly representative of the racial and ethnic demographics of the United States, reflecting the characteristics of the populations served by the participating sites.

In our study, rWGS was ordered in both hospitals that had medical genetics as the primary service implementing rWGS, as well as those that did not. In the latter situation, testing was primarily ordered by intensivists and a few by non-genetic specialists such as cardiologists and neurologists. Across sites, rWGS was ordered for a wide range of phenotypic indications. Our study did not find any statistically significant difference in rate of diagnosis or management change based on the phenotypes reported as the indication for rWGS.

The likelihood of finding a genetic diagnosis using rWGS in this study was similar to many others including NSIGHT1, SouthSeq and Baby Deer, and just below those quoted in Project Baby Bear and GEMINI (7, 13, 14, 16, 18, 21). The likelihood of uncertain results was also similar to that of SouthSeq, which is important to note as that study focused on racial/ethnic minorities and underserved populations, which can result in a higher proportion of results classified as VUS (18). While there are important advantages of WGS over WES, the vast majority of diagnoses identified in this study would likely have been detected using WES, which has been documented in prior studies (13). The simplified library preparation allowed by WGS does reduce the time to diagnosis and likely the clinical utility as a result of quicker turnaround. As improved analytic techniques continue to identify a wider range of genetic mechanisms from WGS data, the difference in diagnostic yield between WGS and WES is likely to grow over time.

Our study found a lower, but still clinically impactful, rate of genetic diagnosis for extremely and very premature infants at 16%, compared to a diagnostic rate of 37% for infants born at 32 weeks gestation or later. Infants with extreme prematurity are more prone to multi-system illnesses such as intraventricular hemorrhages and necrotizing enterocolitis that are generally multifactorial and not monogenic in etiology. Babies admitted to an ICU and born closer to term would not be expected to have such critical illness due to gestational age alone, and therefore the admissions may be more likely due to a monogenic disease. These data differ from the results of the NICUSeq study, which indicated a higher diagnostic rate for their extremely and very premature infants at 28%. However, the NICUSeq study noted that only premature infants with high concern for genetic etiologies were included and varying ascertainment may explain these different results (2). Despite the lower rate of genetic diagnosis found in extremely and very premature infants in our study, rWGS was still found to have a significant impact on medical decision making in that group of patients.

As expected, given the breadth of phenotypes among subjects of this study, most genetic etiologies of disease identified via rWGS were seen in only a single subject (see Supplementary Table S3). The only diagnoses identified in more than one subject were Kabuki syndrome, Prader Willi syndrome, CHARGE syndrome and Schaff-Yang syndrome. The wide diversity of diagnostic results emphasizes the value of broad and hypothesis-free testing as opposed relying on the clinical experience of clinicians to accurately identify rare diseases, especially in infants that lack specific phenotypic features that may develop as part of the natural history of these diseases.

Another notable result from our study was providers’ impression of rWGS impact on medical management. As previously described, obtaining a positive or a negative rWGS result in critically ill infants can impact medical management during a single hospitalization. In our study, 32% of infants were found to have a major medical management change as a result of rWGS results. The fraction of infants with changes in management due to rWGS did not significantly differ based on gestational age, age at testing or phenotypic category. The likelihood of change in medical management in our study was similar to Project Baby Bear, slightly above that of NSIGHT and Baby Deer, and higher than that found in the NICUSeq study (2, 13, 14, 16, 22). In comparing these studies, there was a correlation between faster turnaround time of testing and higher rate of impact on medical management. For this study, the average time to diagnosis was six days as compared to three days in Project Baby Bear and eleven to fifteen days in NSIGHT and NICUSeq. In all cases, time to result was dramatically faster than the many weeks required for genomic testing prior to the development of rapid methods.

Only one previous study has examined the clinical impact of negative rWGS results and none to date have examined the impact of variants of unknown significance. Here we found, in agreement with several previous studies, that diagnostic rWGS results were associated with a high rate of changes in management (97%). We also found that a significant proportion of infants had a change in management following receipt of a negative result (58%). NSIGHT2 was the only prior study to examine the clinical utility of non-diagnostic rWGS results (13). They found that 63% of diagnostic rWGS results were associated with changes in management, whereas 16% of negative rWGS results were associated with changes in management. The definition of change in management used in the NSIGHT2 study was similar to that of major rWGS-associated changes in management herein (changes in medication, diet, surgery, or palliative care). Major changes in management, specifically, were reported in our study for 70% of infants receiving diagnostic rWGS results, and 7% of infants receiving negative rWGS results, which were similar to those of NSIGHT2 (13). No prior study has examined the clinical impact of uncertain rWGS results. From our data, 68% of the infants who received a VUS result had a consequent change in management, of which 23% were major management changes. These data indicate that negative and uncertain results do have clinical utility in some patients. Negative results always lower the posterior probability of a genetic etiology and can refocus testing on non-genetic etiologies. Because of the high sensitivity of WGS for many genetic conditions, negative results can dramatically reduce the probability of some diagnoses for which interventions were being considered or given. Uncertain results can raise the posterior probability of specific genetic disorders and suggest a diagnostic hypothesis that can be assessed with subsequent confirmatory tests. In some cases, clinical interventions with low risk can be implemented on the basis of genomic results classified as a VUS, for example use of vitamins or other enzymatic cofactors. There is significant need for additional study of the clinical utility of negative and uncertain rWGS results and their comparison with lower sensitivity genetic tests such as panel and exome sequencing.

Note that the interpretation of the clinical utility of rWGS in our study was limited by the retrospective and subjective nature of the assessments of changes in medical management, length of stay, and quality of care. Additionally, the ascertainment of patients for rWGS differed between sites based on the local practice environment and specific protocols for testing. However, by aggregating the data from participating sites we aimed to provide additional data on the value of implementation of rWGS for admitted infants that was broadly generalizable to the settings in which many patients around the United States receive care.

Our study did collect a global impression obtained from providers regarding whether the impact of rWGS on clinical care was positive, negative or neutral. For the vast majority of patients, providers reported that their global impression regarding the impact of rWGS was favorable, especially when testing resulted in a genetic diagnosis. This is likely due to the ability to implement more targeted and potentially disease-modifying care for the patients. Providers felt that 20% of patients were likely to have had a shorter hospital stay due to use of rWGS. This provider impression supports one of the conclusions from the NICUSeq study, where they found that early testing and diagnosis allowed for more focused clinical management (2). However, that study did not find a statistically significant difference in length of stay or survival. Further study on that topic is indicated since shorter stay is likely correlated with lower cost of care, supporting additional data from previous studies which have shown that rWGS is likely to be a cost-effective intervention for admitted infants (14, 16). Unlike BabySeq, Project Baby Bear, and Project Baby Manatee, which all projected cost reduction with use of rWGS, our study did not directly assess the economic impact of rWGS (12, 14, 15).

In summary, we document successful implementation of rWGS in the care of a diverse population of admitted infants at eight institutions with variable resources and experience with genetic testing, providing further evidence for the clinical utility of rWGS across a range of inpatient contexts. Additionally, this study suggests clinical utility for negative and uncertain rWGS results in addition to positive results, though additional research is needed on that topic.

## Data Availability

The original contributions presented in the study are included in the article/**Supplementary Material**, further inquiries can be directed to the corresponding author.

## Appendix 1

**Table:**

Major change	Minor change	No change
Based on genetic result, use of disease modifying medication was indicated	Based on genetic result, use of medication for symptomatic or supportive care was indicated	Genetic result not used in clinical decision making in any way
Based on genetic result, a medication was avoided or stopped due to contraindication for that diagnosis	Based on the genetic result, new specialty consultation was obtained	
Based on the genetic result, a potentially disease modifying dietary change was implemented	Based on the genetic result, specialty consultation was no longer required	
Based on the genetic result, a surgery or procedure was indicated	A new imaging study was obtained based on the genetic result	
Based on the genetic result, a surgery or procedure was avoided that was contraindicated for the diagnosis	A previously indicated imaging study was avoided based on the genetic result	
Based on the genetic result, the timing of surgical intervention changed	An additional lab test was obtained based on the genetic result	
A disease-specific screening protocol was implemented based on the genetic result	A previously indicated lab test was avoided based on the genetic result	
Palliative care was offered and/or initiated based on the genetic results	A disease-specific screening protocol was implemented for a family member of the proband based on the genetic result	
The involvement of the palliative care team was ended based on the genetic results	Genetic counseling was provided to the family based on the genetic result	
Organ transplantation was indicated based on the genetic result	An additional family member underwent testing based on the genetic result for the proband	
Organ transplantation was considered to be contraindicated based on the genetic result		
